# Managing benzodiazepine dependence arising from use of the adulterated opioid supply: a commentary

**DOI:** 10.1186/s13011-026-00747-0

**Published:** 2026-07-20

**Authors:** Gurkiran Parmar, Nicole Malette, Josey Ross, Alexis Crabtree, Paxton Bach

**Affiliations:** 1 BC Centre on Substance Use, Vancouver, BC Canada; 2 BC Centre for Disease Control, Vancouver, Canada; 3 BC Centre on Substance Use & UBC Department of Medicine, Vancouver, Canada

**Keywords:** Benzodiazepines, Street drugs, Adulterants, Opioids

## Abstract

The growing presence of benzodiazepine-adulterated opioids in the unregulated drug supply poses significant challenges for clinicians treating people with opioid use disorder. This commentary highlights key areas of uncertainty and emerging agreement on screening, risk assessment, withdrawal timelines, and treatment approaches for individuals using benzodiazepine-adulterated opioids. It also identifies gaps in community-based support, the effectiveness of drug checking, and the role of medications for opioid use disorder in managing opioid-benzodiazepine withdrawal. Together, these scenarios demonstrate rapidly evolving challenges and the need to refine clinical practices in order to improve care for individuals affected by the evolving unregulated drug supply.

## Background

From January 1, 2023, to December 31, 2025, voluntary drug testing results in British Columbia, Canada, showed an increasing prevalence of benzodiazepines in the unregulated drug supply, with nearly 50% of samples testing positive in 2025 [1]. The growing contamination of the drug supply has developed alongside changes in clinical guidance and health system capacity following the declaration of a public health emergency in 2016 [2]. Against the backdrop of a healthcare system already grappling with the rapid emergence of fentanyl and other synthetic opioids dominantating the unregulated opioid supply [2, 3], growing concerns about the risks associated with opioid prescribing led clinicians to adopt more cautious prescribing practices for both opioids and benzodiazepines [4]. In the absence of alternative options, ongoing access to the unregulated market has contributed to an increased, and often inadvertent, frequency of benzodiazepine exposure and physical dependence.

Increased exposure to benzodiazepines through the unregulated drug supply complicate opioid withdrawal management and opioid use disorder (OUD) treatment, as there are no formal guidelines or evidence to support co-withdrawal care currently available [5]. These clinical uncertainties are illustrated in the following vignette: You are a physician or nurse practitioner supporting a patient who is using fentanyl, but has decided to try medications for opioid use disorder (MOUD). Their long-term goal is abstinence from opioids. In previous years, you would only be managing opioid withdrawal during this type of transition. However, you now have to try to determine whether they have been exposed to benzodiazepines and, if so, have developed a significant concurrent physical dependence. If you assume no dependence and are wrong, at best the patient may not find their MOUD as effective at managing cravings and withdrawal, or at worst they could experience a seizure or other harmful withdrawal symptoms. If you incorrectly assume dependence and decide to treat with a benzodiazepine taper, you may contribute to an increased risk of overdose and/or an iatrogenic benzodiazepine dependency. While recommending inpatient care is often the safest approach if benzodiazepine exposure is assumed, it can sometimes discourage individuals from moving forward with treatment, and is impractical in many settings. This example highlights the complex decision-making processes clinicians currently face when managing co-occurring opioid and benzodiazepine withdrawal.

A recent publication from British Columbia, Canada, attempts to establish expert consensus on risk stratification, diagnosis, and treatment for co-occurring opioid and benzodiazepine withdrawal in an environment where close to 50% of the unregulated opioid supply is adulterated with benzodiazepines [1, 6]. However, the realities of the rapidly changing drug supply highlight an urgent need to rethink how we might implement responsive approaches to research and knowledge translation in this evolving space. As the unregulated drug supply continues to change at an unprecedented rate, conventional research and publication timelines (often measured in years) are insufficient to support our ever-changing clinical needs [7]. In the context of an ongoing public health crisis, more timely approaches to research, knowledge synthesis, and dissemination are needed to ensure emerging findings can better inform care. This commentary summarizes some of the remaining unknown elements of risks, diagnosis, and care for individuals experiencing benzodiazepine withdrawal related to unregulated opioid use. By drawing attention to these gaps, we aim to highlight the clinical uncertainty facing providers, identify key research questions, and underscore the urgent need for more attention to this growing problem.

### Risk of benzodiazepine withdrawal

The proportion of unregulated opioids that are testing positive for benzodiazepines is increasing over time. Time-series analysis of opioid samples, from January 1, 2023, to December 31, 2025, has detected monthly values ranging from 20% to 50%, with continued within-month and between month variability in rates of benzodiazepine detection, types of benzodiazepines detected, and quantities detected [1]. And yet, in spite of this prevalence, we are still limited in our assessments with regards to basic questions such as who might be at highest risk of continued exposure. Accessing opioids from the same seller or only using opioids with similar packaging does not guarantee the same contents between purchases. Because the unregulated drug supply is highly variable and substances sold as opioids contain a range of unexpected compounds, all individuals accessing the unregulated drug supply are at increased risk of unintended benzodiazepine exposure [1]. Drug checking programs also routinely find “expectation-discordant” results, meaning substances people thought they had (or did not have) are frequently inconsistent with their chemical analysis [8]. These findings cumulatively demonstrate that asking people who are using unregulated opioids what they are actually being exposed to is insufficient for clinical risk assessment in managing withdrawal.

In the vignette provided above, a UDT might have to be ordered to help distinguish benzodiazepine exposure from non-exposure. However, urine immunoassays provide little information on when the exposure occurred, or how often. The limited sensitivity of urine drug tests for many novel benzodiazepine analogues such as etizolam further constrain clinicians’ abilities to confirm exposure [9].

Lastly, it also remains unclear if people who use available drug checking services have a lower risk of inadvertent benzodiazepine dependence. The effectiveness of drug checking depends on both the type of benzodiazepines and the methods used (e.g., test strips versus Fourier Transform Infrared Spectroscopy [FTIR]) [10]. Work by Laing et al. found that across 159 samples of unregulated opioids in Vancouver, BC, 15.1% contained at least one novel benzodiazepine, with none expected to be positive by the participant [10]. They also noted that false positive and negative rates were 15.5% and 37.5% for test strips, and 3.9% and 91.7% for FTIR, respectively [10]. Although the practice of drug testing is a recognized harm reduction approach, some individuals may falsely assume safety due to potentially inaccurate results. Uncertainty about the prevalence and types of novel benzodiazepines in the unregulated drug supply therefore remains [10] with further research needed to better understand any potential role for drug checking services and technologies in answering this difficult clinical problem.

### Testing for benzodiazepine exposure and withdrawal

Without adequate tools for the reliable diagnosis of benzodiazepine withdrawal in the context of unintended exposure and concurrent opioid use, safe and appropriate initiation of management strategies will remain a challenge. As in the scenario above, a physician or nurse practitioner initiating MOUD for a patient using unregulated opioids must consider whether concurrent benzodiazepine exposure and dependence are present, and missing this risk could lead to harmful withdrawal outcomes. While existing withdrawal assessment tools may still be clinically useful in these situations, they were not specifically designed to identify or distinguish co-occurring benzodiazepine and opioid withdrawal. This can be challenging considering the fact that opioids and benzodiazepine withdrawal syndromes share many overlapping features, including anxiety, agitation, insomnia, gastrointestinal upset, automimic hyperactivity, irritability, and mood disturbance [6]. This diagnostic ambiguity is reflected in outpatient care, inpatient withdrawal management, and even critical care settings, where mixed withdrawal syndromes are increasingly described. The growing presence of alpha agonists, such as medetomidine, in the unregulated supply further complicates assessment and management, as withdrawal symptoms may overlap with or mask those with opioids, benzodiazepines, and other substances.

Urine immunoassays are a diagnostic tool with significant limitations in the diagnosis of benzodiazepine exposure and withdrawal, as noted above. Confirmatory laboratory testing (e.g., gas or liquid chromatography/mass spectrometry) can detect more types of benzodiazepines, but variability in laboratory capacity, turnaround time for results, and cost can further limit the utility in the timely management of withdrawal symptoms. More research is needed to determine what role such a tool can (or cannot) play in the diagnosis of benzodiazepine exposure and dependence, along with how to use this information to inform diagnosis and treatment plans.

Tools such as the Clinical Institute Withdrawal Assessment Scale – Benzodiazepines (CIWA-B) and the Clinical Institute Withdrawal Assessment Scale – Alcohol Revised (CIWA-Ar) have been proposed as potentially useful for this purpose [4]. Both are validated tools to monitor for signs of withdrawal, albeit ones that have not been studied in this unique context. It is important to note, however, that Clinical Institute Withdrawal Assessment (CIWA) results may be influenced by other factors affecting an individual’s withdrawal experience. For example, many of the CIWA items (e.g., anxiety, restlessness, tremors, nausea, sweating) also occur during opioid withdrawal [11]. The overlapping symptoms may make it challenging to distinguish the co-occurrence of benzodiazepine and opioid withdrawal, since the tool was not designed to screen for opioid withdrawal. CIWA-B is theoretically designed to specifically monitor for signs and symptoms of benzodiazepine withdrawal; however, healthcare staff may also be less familiar with the CIWA-B tool compared to CIWA-Ar [12]. Moreover, there is limited guidance on how to adapt existing assessment tools for the realities of the toxic unregulated opioid supply, leaving providers uncertain about how to interpret or utilize the results of the assessment tool. Given these concerns, more work is needed to determine the appropriateness of CIWA scores for assessing patient history and conducting physical examinations in cases of co-occurring benzodiazepine and opioid withdrawal. Developing or adapting standardized tools that account for polysubstance exposure (including the emergence of other adulterants such as alpha agonists in the unregulated drug supply), diverse care settings (e.g., emergency departments, community clinics, harm reduction sites), and varying levels of provider experience will be crucial for improving diagnosis and management practices.

### Care for individuals experiencing benzodiazepine withdrawal

Another knowledge gap is identifying how to incorporate both the known and unknown aspects of benzodiazepine adulteration in the unregulated drug supply into evidence-informed clinical guidelines and treatment planning. While managing benzodiazepine withdrawal in isolation classically requires extended care [6], the appropriate setting and duration for care in this unique context remains unclear and may vary by individual health status and substance use patterns. Novel benzodiazepines may differ considerably in their pharmacokinetics and pharmacodynamics, creating added uncertainty about how long symptoms may persist and how they may present clinically [10]. Further, existing clinical guidance and withdrawal management approaches are primarily based on benzodiazepine withdrawal among individuals who are aware of both the nature and frequency of their benzodiazepine use. More information is required to understand the highest risk period for withdrawal from benzodiazepine-adulterated opioids, how this may vary depending on the dominant benzodiazepine classes found in the drug supply at any given time, the potential clinical course(s), possible differences compared to withdrawal from prescribed benzodiazepines, and the time needed for lingering symptoms to resolve.

MOUD reduce symptoms of opioid withdrawal and appropriate use of MOUD may potentially lessen the complexity of managing concurrent withdrawal symptoms. However, they do not directly address benzodiazepine withdrawal symptoms [13] or exclude someone from exposure to benzodiazepines through continued unregulated opioid use. In the scenario provided, most would agree that initiation and titration of MOUD to a therapeutic dose would be an appropriate initial step in order to manage the known OUD, whether concurrent benzodiazepine dependence is present or not. Further supplementation with unregulated opioids may thus be a result of inadequate MOUD dosing, gaps in access, other difficulties with treatment adherence, or the unrecognized consequences of untreated benzodiazepine withdrawal [13]. There is little evidence about whether clinicians providing MOUD adjust their prescribing or monitoring practices in response to the risks presented by novel benzodiazepine adulteration, or how such adaptations might influence patient outcomes. In these cases, appreciating patient exposure (known or unknown) to benzodiazepines is particularly relevant.

Inpatient care is strongly recommended for any patient determined to be at high likelihood of experiencing concurrent benzodiazepine and opioid withdrawal, and is considered essential for individuals experiencing moderate to severe withdrawal symptoms, those whose symptoms escalate, and those with complicating health conditions that increase clinical risk [6]. Despite this, decision-making around triage and level of care is often nuanced, with approaches varying depending on clinical experience, local protocols, available resources, and the individual complexity of each case. Challenges such as limited availability of services in rural or remote areas, as well as broader system constraints (e.g., bed availability, fragmented pathways between acute and community care) may impact care planning [14]. There also remains a lack of clarity on when and if it is appropriate or possible to manage benzodiazepine withdrawal safely in community. A major limitation in benzodiazepine care is the lack of availably of structured, community-based supports and protocols to guide safe management outside of inpatient settings. Community settings generally do not have the same capacity for around-the-clock medical monitoring. Overall, these factors demonstrate a care gap: inpatient support is needed but limited, while the appropriateness of community-based options remains unclear.

## Conclusions

The current clinical uncertainty around co-occurring opioid and benzodiazepine withdrawal reflects the rapidly evolving and increasingly complex nature of the unregulated drug supply. In BC, previous work has begun a process of understanding and applying consensus-based recommendations for managing benzodiazepine withdrawal in the context of the adulterated drug supply [6]. It also highlighted significant knowledge gaps and areas for further understanding related to this serious health challenge. Further information is needed to better understand risks, effective tools for diagnosing, and appropriate durations of care for individuals experiencing benzodiazepine withdrawal related to the use of unregulated opioids. Despite this work, the composition of the unregulated drug supply continues to change faster than conventional research processes can keep up, limiting our ability to develop evidence-informed clinical guidance that is both accurate and contemporary.

These realities underscore the need for more timely, responsive approaches to understanding this challenging clinical issue. Current methods of translating novel interventions into research development can be glacially slow during periods of urgent need. Traditional research and knowledge translation timelines are deliberate, iterative, and often require years from data collection to dissemination [6]. In the context of a rapidly changing and increasingly toxic unregulated drug supply, this means findings may already be outdated by the time they become available to clinicians. There is a critical need to implement emerging observational and interventional research approaches to promote knowledge translation cycles within 6–12 month periods. Targeted research with shorter knowledge translation cycles will support a more timely, responsive, and ultimately effective reaction to an ongoing public health crisis.

## Data Availability

Not applicable.

## Abbreviations

OUD: Opioid use disorder
MOUD: Medications for opioid use disorder
UDT: Urine Drug Testing
FTIR: Fourier Transform Infrared Spectroscopy
CIWA-B: The Clinical Institute Withdrawal Assessment Scale – Benzodiazepines
CIWA-Ar: Clinical Institute Withdrawal Assessment Scale – Alcohol Revised
CIWA: Clinical Institute Withdrawal Assessment
